# Six weeks of dynamic apnoeic training stimulates erythropoiesis but does not increase splenic volume

**DOI:** 10.1007/s00421-020-04565-5

**Published:** 2020-12-29

**Authors:** Antonis Elia, Matthew J. Barlow, Oliver J. Wilson, John P. O’Hara

**Affiliations:** 1 Division of Environmental Physiology, School of Chemistry, Bioengineering and Health, KTH Royal Institute of Technology, Berzelius väg 13, Solna, 171 65 Stockholm, Sweden; 2 Carnegie School of Sport, Leeds Beckett University, Leeds, UK

**Keywords:** Apnoea, Spleen, Haemoglobin, Erythrocytes, Erythropoietin, Iron

## Abstract

**Purpose:**

This study examined the influence of dynamic apnoea training on splenic volume and haematological responses in non-breath-hold divers (BHD).

**Methods:**

Eight non-BHD performed ten maximal dynamic apnoeas, four times a week for  six weeks. Splenic volumes were assessed ultrasonically, and blood samples were drawn for full blood count analysis, erythropoietin, iron, ferritin, albumin, protein and osmolality at baseline, 24 h post the completion of each week’s training sessions and seven days post the completion of the training programme. Additionally, blood samples were drawn for haematology at 30, 90, and 180 min post session one, twelve and twenty-four.

**Results:**

Erythropoietin was only higher than baseline (6.62 ± 3.03 mlU/mL) post session one, at 90 (9.20 ± 1.88 mlU/mL, *p* = 0.048) and 180 min (9.04 ± 2.35 mlU/mL, *p* = 0.046). Iron increased from baseline (18 ± 3 µmol/L) post week five (23 ± 2 µmol/L, *p* = 0.033) and six (21 ± 6 µmol/L; *p* = 0.041), whereas ferritin was observed to be lower than baseline (111 ± 82 µg/L) post week five (95 ± 75 µg/L; *p* = 0.016), six (84 ± 74 µg/L; *p* = 0.012) and one week post-training (81 ± 63 µg/L; *p* = 0.008). Reticulocytes increased from baseline (57 ± 12 × 10^9^/L) post week one (72 ± 17 × 10^9^/L, *p* = 0.037) and six (71 ± 17 × 10^9^/L, *p* = 0.021) while no changes were recorded in erythrocytes (*p* = 0.336), haemoglobin (*p* = 0.124) and splenic volumes (*p* = 0.357).

**Conclusions:**

Six weeks of dynamic apnoeic training increase reticulocytes without altering mature erythrocyte concentration and splenic volume.

## Introduction

Competitive breath-holding is a popular sport with athletes competing for the longest apnoeic duration they can sustain in a static position or the maximal distance they can cover horizontally/vertically whilst holding their breath with or without fins. In humans, apnoeic capabilities are dictated by: bodily oxygen stores (Elia et al. 2019b), the rate of oxygen conservation and utilisation (Costalat et al. 2017; Ferretti et al. 1991; Lemaitre et al. 2005, 2008), hypoxemic and hypercapnic tolerance (Bain et al. 2016; Taboni et al. 2019; Willie et al. 2015), and training experience including an individual’s psychological tolerance to the increasing urge to breathe (Delapille et al. 2001; Schagatay et al. 2000). Evidence suggests that elite breath-hold divers (EBHD) have a greater oxygen storage capacity in both blood and skeletal muscle tissue (Elia et al. 2019b), a stronger diving reflex response (Costalat et al. 2017; Elia et al. 2020; Lemaitre et al. 2005, 2008), and a greater hypoxemic and hypercapnic tolerance compared with non-divers (ND) (Bain et al. 2016; Elia et al. 2020; Joulia et al. 2002; Willie et al. 2015). Collectively, these physiological characteristics enable EBHD to suppress respiratory urges and sustain apnoeas for extended durations.

To date, there is an abundance of research that has investigated the physiological modifications (i.e. cardiovascular, splenic and haematological responses) during and/or post voluntary apnoeic epochs (Elia et al. 2019a, 2020; Palada et al. 2007; Schagatay et al. 2001, 2005). On the contrary, limited research exists that has assessed the longitudinal effects of apnoeic training (Bouten et al. 2019; Engan et al. 2013; Joulia et al. 2003; Schagatay et al. 2000). Interestingly, static apnoea training (i.e. 2–8 weeks; 5–10 apnoeic bouts per day) has been documented to: (1) augment the magnitude of the diving-reflex-induced bradycardial response (Schagatay et al. 2000), (2) expand splenic volume and (3) enhance resting reticulocyte (Engan et al. 2013) and haemoglobin concentrations (Bouten et al. 2019). In addition, Joulia et al. (2003) demonstrated that three months of simulated dynamic apnoea training (i.e. steady-state cycling combined with apnoeic epochs, three times per week) significantly enhanced hypoxic and hypercapnic tolerance. This suggests that some of the physiological characteristics exhibited by EBHD stem, at least in part, from a training-induced stimulus.

To the best of our knowledge there are no reports addressing the longitudinal effects of dynamic apnoea training on erythrocyte concentrations. Acute bouts of dynamic apnoeas are associated with a stronger hypoxemic stress and a greater post-apnoeic erythropoietic release compared with static apnoeas (Barlow et al. 2018; Elia et al. 2019a; Overgaard et al. 2006). Therefore, since the degree of hypoxemia is directly proportional to the magnitude of EPO release and erythropoiesis (Eckardt et al. 1989; Elia et al. 2019a; Elliott 2008; Jelkmann 2011; Knaupp et al. 1992), it is tempting to contemplate that a training protocol comprising a series of dynamic, rather than static apnoeas, would serve as a stronger stimulus for erythrocyte neoformation. Accordingly, the present study will aim to provide a novel insight to the effect of a six-week dynamic apnoea training protocol on erythrocyte concentrations.

The splenic responses to apnoeic conditions in both trained and untrained breath-hold divers have been examined extensively in the literature (Elia et al. 2020; Palada et al. 2007; Schagatay et al. 2001, 2005). Conversely, the longitudinal effects of apnoeic training on splenic volume have received limited attention (Bouten et al. 2019; Engan et al. 2013). Current evidence signifies that apnoeic training periods lasting more than two weeks are necessary to elicit splenic volume gains (Bouten et al. 2019; Engan et al. 2013). Specifically, Bouten et al. (2019) reported splenic volume gains following four (+ 20%; 47 mL) and eight weeks (+ 24%; 58 mL) of static apnoeic training (i.e. five apnoeic bouts per day) compared to baseline. In addition, Rodriguez et al. (2015) reported significant increases (+ 40%; 77 mL) in basal splenic volumes following six weeks of trekking at high-altitude. These studies signified that repeated hypoxemic exposures and prolonged hypoxic exposures prompt splenic expansion. Although the underlining mechanisms that dictate splenic volume are still under debate, it is reasonable to conjecture that hypoxia/hypoxemia may serve a key role in regulating this. Consequently, a dynamic apnoea training protocol, which facilitates a greater hypoxemic stress than static apnoeas, may provide a stronger stimulus for splenic growth.

Accordingly, the aims of the present study were to provide a novel insight to the erythropoietic effects of a six-week dynamic apnoea training program by assessing a comprehensive panel of haematological markers (i.e. full blood count analysis, EPO, iron, ferritin and plasma osmolality, protein and albumin), and to investigate whether such regimen is capable of inducing splenic growth. We hypothesised that six weeks of dynamic apnoea training performed by ND will upregulate resting erythrocyte concentrations and will induce splenic volume expansion.

## Materials and methods

### Participants

Eight male participants volunteered for this study (height, 178 ± 1 cm; body mass, 76 ± 5 kg; age, 24 ± 4 years). Participants were healthy, non-smoking, physically active (i.e. accumulated at a minimum 30 min of moderate intensity of physical activity per day [activities included walking, jogging, futsal and rugby], and did not participate in any competitive form of sport) and provided written informed consent prior to participating in the study. The study was granted ethical approval by the Leeds Beckett University ethics committee (Ethical Approval Number 36538) and all experimental procedures were conducted in accordance with the Declaration of Helsinki.

### Experimental protocol

**Fig. 1:**
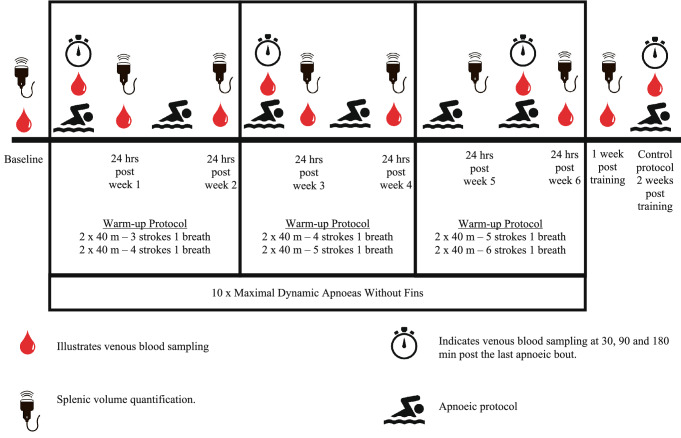
Schematic representation depicting the data collection time points and the six-week apnoeic training protocol

The present study was divided into four parts (i.e. part 1, Preliminary Measurements [Week 1]; part 2, Familiarisation [Week 1]; part 3, Training Program [Weeks 2–8]; part 4, Control Protocol [Week 9]) which were all completed within nine weeks (Fig. 1). No iron supplementation was used by any of the participants for three months prior to, and during the study. Additionally, participants were instructed to maintain a consistent diet throughout the study. During each testing day participants reported at Leeds Beckett University after a 12 h fast and abstinence from caffeine and alcohol containing beverages. In addition, participants were instructed to refrain from physical activity for 24 h prior to and during each testing day.

### Preliminary measures

Following arrival at the laboratory (~ 25 °C), the participant’s anthropometric measurements including stature and weight were assessed (Seca, Vogel & Halke, Hamburg, Germany). Thereafter, they underwent a 20 min supine resting period following which baseline splenic volumes were then quantified using a non-invasive ultrasonic portable device (MindRay DP-50, Shenzhen Mindray Bio-Medical Electronics Co., Ltd., Shenzhen, China) using a technique described in detail elsewhere (Elia et al. 2019a, 2020). Briefly, participants were seated vertically while the site for spleen measurements was identified from the dorsal side. Three measurements of each triaxial measurement point of the spleen’s maximal length (*L*), thickness (*T*) and width (*W*) were determined (coefficient of variation [CV] ~ 6%), with the mean for each point being used to calculate splenic volume through the use of the Pilström formula (*L*π[*WT* − *T*^2^]/3). Thereafter three whole blood samples were drawn from a suitable vein in the antecubital fossa of the participant’s arm (median cubital vein and basilic vein) to assess circulating serum concentrations of EPO, iron, ferritin, osmolality, albumin, protein (2 × 6 mL; BD Vacutainer, 367954, Plymouth, UK) and for a full blood count analysis (i.e. reticulocytes, erythrocytes, haemoglobin, haematocrit, mean cell volume, mean cell haemoglobin and red blood cell distribution width) (4 mL; BD Vacutainer, K2E EDTA, BD, Plymouth, UK) to be performed.

### Familiarisation session

Within 24 h of completing the preliminary measures participants reported at the Leeds Beckett University swimming facilities (~ 28 °C) and a familiarisation session was performed. Participants were introduced to the dynamic apnoea technique (horizontal underwater breast-stroke swimming) and were familiarised to the trial conditions and requirements.

### Dynamic apnoea training program

The program required participants to attend once a day for four consecutive training sessions per week, for a total of ix weeks (totalling 24 training sessions) with each session approximately lasting ~ 50 min (Fig. 1). Each training session commenced with four repetitions of 40 m breast-stroke, serving as a warm-up, with a progression in the number of strokes per breath every two weeks (Fig. 1). Each repetition was separated by a 2 min resting period whereby the participants were allowed to relax and breathe normally. Following completion of the warm-up regime, the dynamic apnoea protocol was initiated.

### Dynamic apnoea protocol

Participants performed ten supervised maximal dynamic apnoeas with each repetition being separated by a 2 min rest period, during which time they were allowed to relax and breathe normally whilst remaining immersed in water. Participants were instructed to hold their breath without prior hyperventilation or lung packing, after a deep but not maximal inspiration. A one minute warning was provided prior to commencing each apnoea, participants received a nose clip 30 s prior to the apnoea (i.e. to reduce any oxygen or water inspiration or oxygen loss) and a 10 s countdown was provided prior to immersing in water and commencing each maximal attempt. During each maximal apnoeic attempt, the time and distance covered was recorded to quantify any changes in performance.

At completion of sessions one (week 1), twelve (week 3) and twenty-four (week 6) a catheter was placed on a suitable vein of the antecubital fossa area of the participant’s arm and one 6 mL whole blood sample was drawn at 30, 90 and 180 min (totalling three 6 mL samples; BD Vacutainer, 367954, Plymouth, UK) post the last apnoea to assess for the level of circulating concentrations of EPO, iron, ferritin, osmolality, albumin and protein. Additionally, 24 h post the completion of each week’s training protocol and one week post the six-week training program the participant’s splenic volumes were quantified, and blood samples were drawn for haematology replicating the preliminary session measurements.

### Control protocol

To control for any possible effects of the warm-up protocol on the circulating serum EPO concentration, seven out of the eight participants repeated the week 5–6 warm-up protocol—as this was the highest warm-up intensity utilised in the present training program. At completion of the control protocol one 6 mL blood sample was drawn at 30, 90, 180 min and 24 h (totalling four 6 mL samples; BD Vacutainer, 367954, Plymouth, UK) post the last repetition to assess for the concentration of circulating EPO.

### Blood sample treatment and analysis

Samples for EPO, iron, ferritin, osmolality, albumin and protein were gently inverted, allowed to coagulate at room temperature for 20 min, and centrifuged (ALC Multispeed Refrigerated centrifuge, PK131R, London, United Kingdom) at 4000 rpm for 10 min at 4 °C. Samples were then aliquoted into Eppendorf tubes and stored at − 80 °C until subsequent analysis. Serum EPO concentrations were quantified using an enzyme-linked immunosorbent assay analysis (R&D systems, Quantikine IVD ELISA, Human Erythropoietin, DEP00, sensitivity 0.6 mIU/mL; CV ~ 3.6%), serum ferritin concentrations were assessed through the use of a two-site immunoenzymatic sandwich assay (UniCel Sxl 800 Access Immunoassay System, Beckman Coulter, London, UK; CV ~ 10%), the phenanthroline method was used to determine serum iron concentrations (AU5800 Series Chemistry Analyzers, Beckman Coulter, London, UK; CV ~ 2.2%), serum albumin was assessed through the bromocresol green method (AU5800 Series Chemistry Analyzers, Beckman Coulter, London, UK; CV ~ 2.1%), serum total protein was evaluated through the biuret method (AU5800 Series Chemistry Analyzers, Beckman Coulter, London, UK; CV ~ 1.5%) and serum osmolality was quantified by a standard freezing point depression technique (3320 Single-Sample Micro Osmometer, Advanced Instruments, Norwood, USA; CV ~ 0.6%). For a full blood count analysis, samples were gently inverted and were analysed within 6 h of collection (Advia 2120i Haematology System, Siemens Healthcare, Surrey, UK; intra-assay variability ~ 5%).

### Statistical analysis

All data were statistically analysed using the IBM SPSS statistics software version 21. Due to technical difficulties, week five's full blood count samples were not analysed. The Shapiro–Wilk test was used to assess whether data were normally distributed (*p* < 0.05). Sphericity was assessed using Mauchly’s test of sphericity; where the assumption of sphericity was violated, the Greenhouse–Geisser correction was applied. Repeated measures ANOVA with post hoc Bonferroni contrast comparisons were used to assess for differences between resting baseline measurements and other collection time points for distance covered, apnoeic duration, EPO, iron, ferritin, reticulocytes, reticulocyte absolute count, erythrocytes, haemoglobin, haematocrit, osmolality, albumin and protein. Friedman test was used to assess the differences within resting baseline measurements and other collection time points for mean cell volume, mean cell haemoglobin and red blood cell distribution width. Where appropriate, partial eta square (*η*^2^) and power (*β*) were also presented. Data are reported as means ± SD and significance was accepted at *p* < 0.05 and *p* = 0.000 was reported as *p* < 0.001. GraphPad Prism version 7.0c was used to construct figures and Word 2016 was used for constructing schematic representations and tables.

## Results

**Fig. 2:**
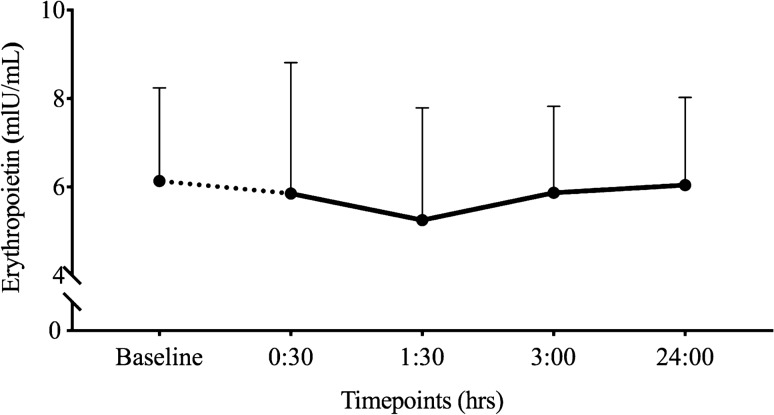
Mean (± SD) EPO (mlU/mL) concentrations from baseline to 24 h post the control protocol

No differences in serum EPO concentrations were observed from baseline (6.14 ± 2.10 mlU/mL) at 30 min (5.85 ± 2.96 mlU/mL), 1 h and 30 min (5.25 ± 2.54 mlU/mL), 3 h (5.87 ± 1.96 mlU/mL) and 24 h (6.04 ± 1.98 mlU/mL) post the control protocol (*p* = 0.251, *η*^2^ = 0.208, *β* = 0.240) (Fig. 2).

### Performance parameters

Mean underwater distance covered and dynamic apnoea time increased from week one to week six by 48% (*p* = 0.004, *η*^2^ = 0.618, *β* = 0.929) and 52% (*p* = 0.010, *η*^2^ = 0.508, *β* = 0.996), respectively (Table 1).

**Table 1:** Mean (± SD) dynamic apnoea performance parameters during the six-week training program

Parameters	Weeks					
	1	2	3	4	5	6
Distance (m)	25 ± 10	29 ± 10	32 ± 9*	32 ± 8*	36 ± 8*	37 ± 7*
Time (s)	29 ± 11	32 ± 9	39 ± 7*	36 ± 9	41 ± 7*	44 ± 7*

*Denotes significance (*p* < 0.05) from week one

### Erythropoietin

**Fig. 3:**
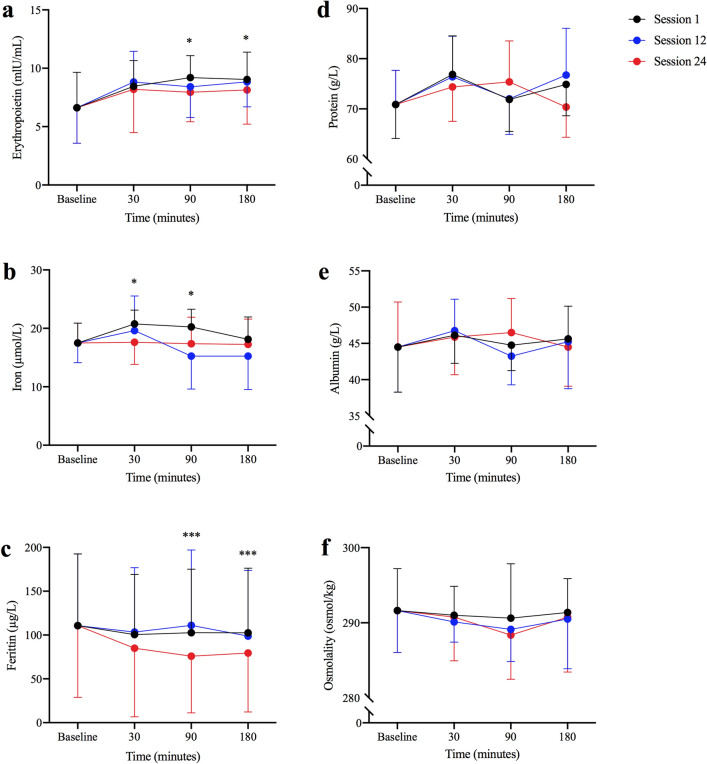
Haematological indices from baseline to 180 min post apnoeas during session one, twelve and twenty-four. Significance from baseline for session one is denoted as *(*p* < 0.05) and for session twenty-four is denoted as ***(*p* < 0.05)

Mean post-apnoeic EPO concentrations were significantly different from baseline post session one (*p* = 0.019, *η*^2^ = 0.335, *β* = 0.774), but not sessions twelve (*p* = 0.209, *η*^2^ = 0.187, *β* = 0.248) or twenty-four (*p* = 0.433, *η*^2^ = 0.084, *β* = 0.123) (Fig. 3a). Specifically, EPO was 39% higher than baseline (6.62 ± 3.03 mlU/mL) post session one at 90 min (9.20 ± 1.88 mlU/mL, *p* = 0.048) and 37% higher at 180 min post-apnoeas (9.04 ± 2.35 mlU/mL, *p* = 0.046), but were not different at 30 min post-apnoeas (8.46 ± 2.21 mlU/mL, *p* = 0.109) (Fig. 3a). Mean post-apnoeic EPO concentrations were not different when compared between sessions (*p* = 0.774, *η*^2^ = 0.021, *β* = 0.086). When post-apnoeic EPO concentrations were expressed as a delta percentage change from baseline, significance was denoted post-session one (30 min, + 69%; 90 min, + 72%; 180 min, + 68%; *p* = 0.033) with a trend being observed post-session twelve (30 min, + 80%; 90 min, + 64%; 180 min, + 68%; *p* = 0.067) but not twenty-four (30 min, + 52%; 90 min, + 58%; 180 min, + 63%; *p* = 0.160).

### Iron

Mean post-apnoeic iron concentration was significantly different from baseline during session one (*p* = 0.003, *η*^2^ = 0.486, *β* = 0.940). Iron increased by 21% from baseline (18 ± 3 µmol/L) at 30 min (21 ± 2 µmol/L, *p* = 0.032) and 17% higher at 90 min (20 ± 3 µmol/L, *p* = 0.014), but no difference was observed at 180 min post-apnoeas (18 ± 4 µmol/L, *p* = 1) (Fig. 3b). Conversely, iron concentrations during the twelth (*p* = 0.345, *η*^2^ = 0.143, *β* = 0.269) and twenty-fourth session (*p* = 0.997, *η*^2^ = 0.002, *β* = 0.052) were not different from baseline, and there was no between-session difference in post-apnoea iron concentrations (*p* = 0.221, *η*^2^ = 0.138, *β* = 0.313) (Fig. 3b). In addition, mean post-apnoeic iron concentrations were significantly different from baseline (*p* = 0.033, *η*^2^ = 0.383, *β* = 0.667), 24 h post week four (− 16%, *p* = 0.048), five (+ 36%, *p* = 0.033) and six (+ 20%, *p* = 0.041) (Table 2).

**Table 2:** Mean (± SD) haematological concentrations during the eight-week period

Variable	Baseline	Weeks						
		1	2	3	4	5	6	One week post
Iron (µmol/L)	18 ± 3	17 ± 3	17 ± 1	19 ± 4	15 ± 3*	23 ± 2*	21 ± 6*	17 ± 6
Ferritin (µg/L)	111 ± 82	112 ± 80	101 ± 71	100 ± 68	101 ± 73	95 ± 75*	84 ± 74*	81 ± 63*
Osmolality (osmol/kg)	292 ± 6	292 ± 7	291 ± 4	288 ± 5	290 ± 4	292 ± 6	293 ± 7	290 ± 6
Albumin (g/L)	45 ± 6	43 ± 7	45 ± 9	41 ± 6	40 ± 10	46 ± 5	46 ± 4	44 ± 5
Protein (g/L)	71 ± 7	67 ± 9	70 ± 13	66 ± 5	63 ± 14	72 ± 5	73 ± 3	71 ± 5
Reticulocytes (%)	1.16 ± 0.18	1.38 ± 0.23	1.13 ± 0.21	1.31 ± 0.15	1.28 ± 0.17	–	1.49 ± 0.33*	1.14 ± 0.31
Reticulocyte absolute count (10^9^/L)	57 ± 12	72 ± 17*	56 ± 9	65 ± 5	63 ± 9	–	71 ± 17*	56 ± 18
Erythrocytes (10^12^/L)	5.02 ± 0.32	4.90 ± 0.33	5.01 ± 0.49	4.88 ± 0.32	5.07 ± 0.43	–	4.93 ± 0.26	4.97 ± 0.33
Haemoglobin (g/dL)	152 ± 8	148 ± 5	150 ± 8	146 ± 8	150 ± 8	–	150 ± 6	148 ± 9
Haematocrit (%)	45 ± 2	44 ± 2	45 ± 2	44 ± 2	46 ± 4	–	44 ± 1	44 ± 2
Mean cell volume (fl)	87 ± 3	89 ± 4	90 ± 5	90 ± 4	90 ± 3	–	89 ± 4	89 ± 3
Mean cell haemoglobin (pg)	30 ± 2	30 ± 2	30 ± 2	30 ± 2	30 ± 2	–	31 ± 2	30 ± 2
Red blood cell distribution width (%)	13.2 ± 0.5	13.2 ± 0.6	13.1 ± 0.6	13.2 ± 0.8	12.9 ± 0.2	–	12.9 ± 0.4	13.0 ± 0.4

Data are presented as mean ± SD

*Denotes significance (*p* < 0.05) from baseline

### Ferritin

Mean post-apnoeic ferritin concentration was significantly different from baseline post session twenty-four (*p* = 0.003, *η*^2^ = 0.614, *β* = 0.946) but not post session one (*p* = 0.129, *η*^2^ = 0.232, *β* = 0.463) and twelve (*p* = 0.70, *η*^2^ = 0.280, *β* = 0.576) (Fig. 3c). During session twenty-four, ferritin was lower than baseline (111 ± 82 µg/L) by 24% at 90 min (76 ± 65 µg/L, *p* = 0.035) and 29% at 180 min post-apnoeas (80 ± 67 µg/L, *p* = 0.033) but no difference was revealed at 30 min post-apnoeas (85 ± 78 µg/L, *p* = 0.100) (Fig. 3c). Moreover, no significant differences were observed when post-training ferritin concentrations were compared between sessions (*p* = 0.769, *η*^2^ = 0.025, *β* = 0.087) (Fig. 3c). In addition, mean ferritin was significantly reduced from baseline (*p* = 0.001, *η*^2^ = 0.505, *β* = 0.973), 24 h post week five (− 10%; *p* = 0.016), six (− 22%; *p* = 0.012) and one week post the completion of the six-week training program (− 20%; *p* = 0.008) (Table 2).

### Protein, albumin and osmolality

Mean post-apnoeic protein (Fig. 3d), albumin (Fig. 3e) and osmolality concentrations (Fig. 3f) were not different from baseline during sessions one, twelve and twenty-four (*p* = 0.083) or when compared between sessions (*p* = 0.53) (Fig. 3). Similarly, protein, albumin and osmolality concentrations were not different from baseline during the six-week training protocol (*p* = 0.187) (Table 2).

### Full blood count analysis

Reticulocyte concentrations significantly increased from baseline 24 h post week six (+ 28%; *p* = 0.020), with concentrations being restored to baseline one week post the completion of the six-week training program (*p* = 0.023, *η*^2^ = 0.284, *β* = 0.824) (Table 2). In addition, reticulocyte absolute count increased significantly from baseline 24 h post week one (+ 26%, *p* = 0.037) and week six (+ 24%, *p* = 0.021) (*p* = 0.018, *η*^2^ = 0.295, *β* = 0.847) (Table 2). There were no main effects of the six-week dynamic apnoea training program on mean erythrocyte (*p* = 0.336, *η*^2^ = 0.144, *β* = 0.411) and haemoglobin concentrations (*p* = 0.124, *η*^2^ = 0.204, *β* = 0.606) or haematocrit (*p* = 0.237, *η*^2^ = 0.167, *β* = 0.486), mean cell volume (*χ*^2^[6] = 9.337, *p* = 0.156), mean cell haemoglobin (*χ*^2^[6] = 6.910, *p* = 0.329) and red blood cell distribution width (*χ*^2^[6] = 2.563, *p* = 0.861) at the different time points from baseline (Table 2).

### Splenic volume

**Fig. 4:**
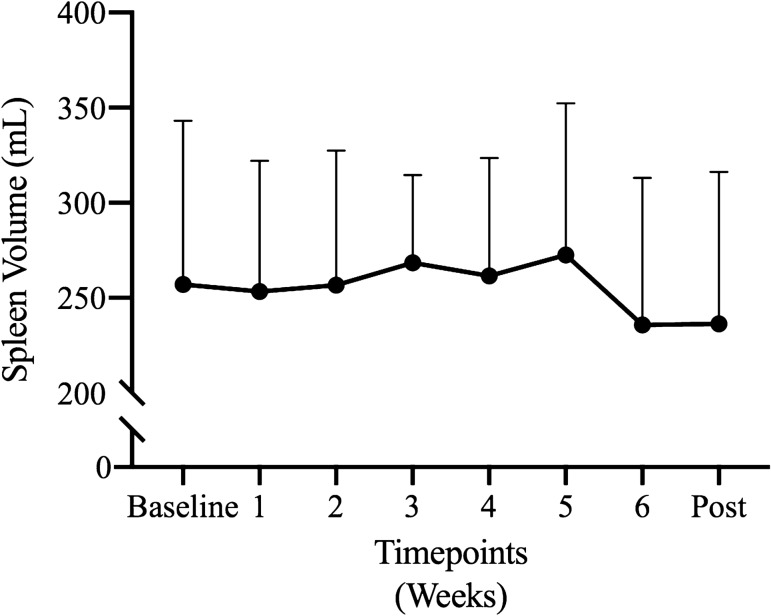
Mean (± SD) splenic volumes during the eight-week period

There was no effect of the six-week training intervention on resting splenic volumes during, and one week after the completion of training (*p* = 0.357, *η*^2^ = 0.140, *β* = 0.273) (Fig. 4).

## Discussion

The present study examined the effect of a six-week dynamic apnoea training program on erythrocyte concentration and splenic volume. The primary findings signify that six weeks of dynamic apnoea training (1) improves apnoeic performance, (2) increases reticulocytes, (3) but does not enhance mature erythrocyte concentration or splenic volume. Collectively, the present study demonstrates that six weeks of dynamic apnoea training activates the process of erythropoiesis, but does not facilitate splenic volume expansion.

### Acute post-apnoeic responses

To the best of our knowledge, this is the first study to assess the effect of ten repeated maximal dynamic apnoeas on EPO concentration acutely and following repetitive daily exposures. Our findings revealed significant increases in EPO following session one, but not post session twelve or twenty-four. These increases are likely caused by the apnoea-induced hypoxemia and not due to haemoconcentration (Cahan et al. 1992; Klausen et al. 1996), since no differences were detected in serum osmolality, albumin or protein concentrations from baseline. Therefore, our study demonstrates that, at least in non-BHDs, ten maximal dynamic apnoeic repetitions are effective in acutely upregulating systemic EPO concentration. However, somewhat counterintuitively, post-apnoeic EPO concentration was not different to baseline with training, despite significant improvements in apnoeic performance. This novel observation might suggest that apnoeic training, similarly to endurance-type exercise training, may attenuate the reduction of renal vascular blood flow (Armstrong and Laughlin 1984; Chen et al. 2001, 1999; De Moraes et al. 2004; DiCarlo and Bishop 1990; DiCarlo et al. 1997; Moyna and Thompson 2004; Mueller et al. 1998; Musch et al. 1991; Yen et al. 1995), subsequently suppressing the release of EPO in response to an acute bout of repeated dynamic apnoeas. However, further research is necessary to determine the extent to which our findings are the result of a training-induced adaptation of the renal vasculature.

Serum iron was markedly elevated at 30 min (+ 21%) and 90 min (+ 17%) post the last apnoeic bout following session one. Our findings are in agreement with earlier studies that reported similar acute increases in iron concentrations following exercise interventions (Peeling et al. 2009a, b, c, 2014; Schumacher et al. 2002). The transient increases in iron may reflect haemolysis, a process whereby an erythrocyte’s membrane is damaged, causing it to release iron and its associated haemoglobin into the extracellular fluid—consequently reducing its lifespan (Buchman et al. 1998; Peeling et al. 2009a, c; Selby and Eichner 1986; Smith 1995; Telford et al. 2003). Oxidative stress has been documented to incite haemolysis and to dictate the magnitude of this response in a dose-dependent manner; with considerable evidence indicating that oxidative damage may be the primary mechanism by which erythrocytes age (Clark 1988; Mohanty et al. 2014; Pigeolet and Remacle 1991; Seppi et al. 1991; Smith 1995; Telford et al. 2003). Interestingly, repeated maximal apnoeic epochs have been evidenced to upregulate the production of reactive oxygen species (ROS) (Joulia et al. 2002, 2003) and aggravate systemic oxidative stress levels (Rousseau et al. 2006; Sureda et al. 2004, 2015). Therefore, the presently recorded marked increases in circulating iron concentrations following session one may be indicative of an oxidative stress-induced haemolysis (Reardon and Allen 2009; Reeder and Wilson 2005; Schümann et al. 2005). In contrast, no changes were observed in iron following session twelve and twenty-four. Considering that long-term apnoeic training lowers post-apnoeic oxidative stress (Joulia et al. 2003; Sureda et al. 2015), it is tempting to speculate that the lack of iron changes following session twelve and twenty-four may, at least in part, relate to a training-induced adaptation that lowers the degree of oxidative stress-induced haemolysis. Howbeit, it is imperative that additional research is conducted to fully elucidate the underlining mechanisms that dictated the presently recorded iron fluctuations.

### Week-by-week responses

This is the first study to examine erythropoietic markers across an apnoeic training program. Reticulocyte concentration was significantly elevated (+ 26%) 24 h post week one Although our findings testify to an augmented erythropoietic process (Guyton and Hall 2006; Mairbaurl and Weber 2012), similarly to Engan et al. (2013), we failed to record any increases in resting erythrocyte and haemoglobin concentrations. Considering that apnoea-induced oxidative stress may activate haemolysis, we propose that the reticulocyte increases recorded 24 h post-week one may be a compensatory response to replenish damaged/old erythrocytes and restore or sustain normal erythrocyte concentration.

Iron concentration was markedly reduced (− 16%) 24 h post week four. These results were likely caused by the apnoeic intervention and not due to diurnal variations, as samples were collected at similar timepoints across the training program. In addition, since no changes were denoted in serum protein, albumin and osmolality concentrations, the reduction in iron did not stem from haemoconcentration or plasma volume fluctuations. Our observations are in agreement with studies that examined iron concentrations 24 h following walking (Terink et al. 2018), marathon (Roecker et al. 2005) and ultramarathon (Chiu et al. 2015) interventions. However, the physiological relevance of these reductions is presently unclear. There is evidence suggesting that iron is implicated in the generation of ROS via the Fenton reaction (Bystrom et al. 2014; Reeder and Wilson 2005), with a number of studies denoting significant increases in oxidative stress and inflammatory responses following excessive iron supplementation (Reardon and Allen 2009; Reeder and Wilson 2005; Schümann et al. 2005). Thus, a reduction in serum iron may be indicative of a bodily response to lower the generation ROS (Liu et al. 2006). On the other hand, a reduction in iron may be indicative of an augmented erythropoietic process. During erythropoiesis, iron demand increases by the bone marrow in order to synthesize heme (Bunn 2013; Muckenthaler et al. 2017; Semenza and Wang 1992). This necessitates an increase in intestinal iron uptake and serum iron binding capacity, as well as enhanced mobilization of iron from internal stores (e.g. ferritin) (Muckenthaler et al. 2017). The availability of sufficient amounts of iron is of critical importance for normal and stress-induced erythropoiesis. Thus, the reductions in iron concentration may be indicative of an increased iron uptake by the bone marrow in response to erythropoiesis. However, further research is required to unveil the physiological relevance of these reductions and to ascertain or refute this hypothesis.

Twenty-four hours post week five and six, we recorded an increase in iron (+ 36% and + 20%, respectively) and a concomitant reduction in ferritin (− 10% and − 22%, respectively) concentration. The marked increases in iron concentration following week six may testify to an enhanced mobilisation of iron from internal stores. Ferritin is the primary iron storage protein, thus serving as a marker of systemic iron deposits (Major et al. 1997; Muckenthaler et al. 2017; Skikne and Cook 1992). As a labile form of iron storage, ferritin makes its stored iron easily accessible during high iron demands (Brugnara et al. 1994a, b; Major et al. 1997). Thus, the observed reductions in ferritin concentration following week six may be indicative of a bodily response to an enhanced iron demand by the bone marrow, increasing the iron availability in response to an augmented erythropoietic process. These fluctuations in iron and ferritin concentrations may suggest that the undulations in EPO (i.e. mean increases of up to 2.21 mlU/mL) observed post session twenty-four, although not statistically significant, may be of physiological significance. Indeed, this hypothesis may partially be supported by the significant increases in reticulocyte count (+ 24%) recorded 24 h post week six. Hence, collectively our novel findings entail that six weeks of dynamic apnoeic training are effective in inciting erythropoiesis.

Erythrocytes remained unchanged throughout the six week training period. Although our study was associated with an increased reticulocyte concentration, the lack of an increase in the erythrocytes and haemoglobin concentrations suggests that longer training periods (e.g. > 6 weeks) and/or greater apnoeic repetitions (e.g. > 10 repetitions) may be necessary for the full cycle of erythropoiesis to be completed. Moreover, one week post the completion of the training period ferritin was the only marker that was not restored to pre-training values, thus providing further evidence to support that longer apnoeic training periods maybe necessary to elicit any significant increases in erythrocytes. Accordingly, future research should investigate the efficacy of longer training durations (e.g. 8–12 weeks) to unveil whether the presently augmented erythropoietic markers translate into new erythrocytes. Additionally, in the present study we did not record any changes in serum osmolality, protein and albumin concentrations, suggesting that our findings were not influenced by blood and plasma volume changes. Future research should seek to also examine total haemoglobin mass to fully elucidate the erythropoietic effects of apnoeic training.

The present study demonstrated that six weeks of dynamic apnoea training were not effective in evoking splenic growth (Fig. 3). Our findings are in agreement with Engan et al. (2013, but are contrary to Bouten et al. (2019) who observed splenic volume gains following eight weeks of static apnoeic training. The underlining mechanisms that dictate splenic volume are currently unclear, however it may be governed by a complex interplay between genetic predispositions and prolonged exposures to hypoxic/hypoxemic conditions (Rodriguez et al. 2015; Ilardo et al. 2018; Bouten et al. 2019). However, since neither us nor Bouten et al. (2019) evaluated the end-apnoeic arterial oxygen saturation levels during the training period, we are currently unable to provide further reasoning to our current findings. Nevertheless, our study may suggest that longer apnoeic training interventions (e.g. > 6 weeks) and/or further training sessions (e.g. daily) may be necessary to facilitate splenic growth.

### Methodological considerations

Our study has a number of methodological considerations. Firstly, although we instructed our participants to maintain a consistent diet throughout the study, we did not evaluate their daily food intake. However, our participants’ iron and ferritin concentrations followed a similar fluctuation trend across the training regimen, which conjointly suggest that our findings stemmed from the prescribed training intervention rather from sudden shifts towards iron-rich/deficient diets. Secondly, although we did not recruit a control group, we did implement several precautionary measures to control against any possible confounders on haematology, including, (i) all blood samples being collected at similar timepoints across the study’s duration, (ii) assessing markers of hydration status, plasma and blood volume, (iii) instructing participants to maintain a consistent diet and (iv) to refrain from any other physical activities during the time course of the study.

### Limitations

In this study, we did not evaluate the magnitude of the hypoxemic stress in terms of arterial oxygen saturation. It is well accepted that hypoxemia is a potent stimulus for erythropoiesis (Eckardt et al. 1989), while recent evidence suggest that it may also promote splenic growth (Rodriguez et al. 2015). It may well be that our study exposed our participants to a lower hypoxemic stress than other studies (Rodriguez et al. 2015; Bouten et al. 2019). If this was so, it would mean that, in non-divers, we do not need a very strong hypoxemic stimulus to induce erythropoiesis. On the other hand, this might further explain the lack of changes in splenic volume in our study. A measure of arterial oxygen saturation would have opened the way to further reasoning about our findings.

## Conclusion

In conclusion, this is the first study to investigate the efficacy of a six-week dynamic apnoea training on haematological indices and splenic volumes. This study highlighted that dynamic apnoeic training increases reticulocyte concentrations without altering mature erythrocytes or splenic volume.

## Abbreviations

ANOVA: Analysis of variance
BHD: Breath-hold divers
CV: Coefficient of variation
EBHD: Elite breath-hold divers
EPO: Erythropoietin
ND: Non-divers
NO: Nitric oxide
ROS: Reactive oxygen species
